# Supplementary material for: UVESCREEN1: A randomised feasibility study of imaging-based uveitis screening for children with juvenile idiopathic arthritis- Study Protocol
Source: PLoS One. 2025 Feb 12;20(2):e0316410. doi: 10.1371/journal.pone.0316410 (PMC11819525; doi:10.1371/journal.pone.0316410)
Supplement: Supplemental material 1 — (DOCX) [file pone.0316410.s001.docx]

Optovue and Heidelberg standardised image acquisition protocols

|  |  | Low volume scan | High volume scan |
| --- | --- | --- | --- |
| Acquisition device |  |  |  |
|  | Manufacturer | Heidelberg | Heidelberg |
|  | Version / Software version | Heidelberg Engineering GmbH | Heidelberg Engineering GmbH |
|  | Device type (time/spectral domain, swept-source, adaptive optics) | software version 6.5; | software version 6.5; |
| Acquisition settings |  | Spectral domain | Spectral domain |
|  | Pupils dilated before examination (y/n) |  |  |
|  | Number of operators and devices | Pupils not dilated | Pupils not dilated |
|  | Background illumination | 1-2 operators, 1 device | 1-2 operators, 1 device |
| Scanning protocol |  |  |  |
|  | Type of scan (circular, volume, star, line, other) | Cross (2xlines) | Volume |
|  | Location | Anterior chamber | Anterior chamber |
|  | Scan parameters (with or without eye tracking) | No eye tracking | No eye tracking |
|  | Volume scan: size of scan, area and location of measurement (degrees or millimeters), number of B-scans, alignment of B-scans, number of A-scans per B-scan | N/A | 12x6mm (height width), 513 A scans / 20 B scans (horizontal) |
|  | Line scan: angle, location, number of A-scans | 12mm length (horizontal and vertical), 513 A scans / 12 B scans | N/A |
|  | Minimum image quality signal strength index | n/a | n/a |

Analysis processes:

(1) Assessed for quality: all images moderate or good, otherwise retaken

(2) Manual identification of hyper-reflective particles indicative of inflammatory cells: >1 in any one image and median of 1 or more across volume scan indicative of ‘positive’ AS-OCT test

|  |  | Low volume scan | High volume scan |
| --- | --- | --- | --- |
| Acquisition device |  |  |  |
|  | Manufacturer | Optovue | Optovue |
|  | Version / Software version | RTVue XR 100 Avanti | RTVue XR 100 Avanti |
|  | Device type (time/spectral domain, swept-source, adaptive optics) | Spectral domain | Spectral domain |
| Acquisition settings |  |  |  |
|  | Pupils dilated before examination (y/n) | Pupils not dilated | Pupils not dilated |
|  | Number of operators and devices | 2 operators, 1 device | 2 operators, 1 device |
|  | Background illumination | Darkened room, illumination not measured | Darkened room, illumination not measured |
| Scanning protocol |  |  |  |
|  | Type of scan (circular, volume, star, line, other) | Cross (2xlines) | Volume |
|  | Location | Anterior chamber | Anterior chamber |
|  | Scan parameters (with or without eye tracking) | No eye tracking | No eye tracking |
|  | Volume scan: size of scan, area and location of measurement (degrees or millimetres), number of B-scans, alignment of B-scans, number of A-scans per B-scan | N/A | 12x6mm (height width), 513 A scans / 20 B scans (horizontal) |
|  | Line scan: angle, location, number of A-scans | 12mm length (horizontal and vertical), 513 A scans / 12 B scans | N/A |
|  | Minimum image quality signal strength index |  | 30 |

Analysis processes:

(1) Assessed for quality: all images moderate or good, otherwise retaken

(2) Manual identification of hyper-reflective particles indicative of inflammatory cells: >1 in any one image and median of 1 or more across volume scan indicative of ‘positive’ AS-OCT test
