# Supplementary material for: UVESCREEN1: A randomised feasibility study of imaging-based uveitis screening for children with juvenile idiopathic arthritis- Study Protocol
Source: PLoS One. 2025 Feb 12;20(2):e0316410. doi: 10.1371/journal.pone.0316410 (PMC11819525; doi:10.1371/journal.pone.0316410)
Supplement: Supplemental material 2 — (DOCX) [file pone.0316410.s002.docx]

**Supplemental material 2- Specific visual acuity screening protocol**

| **Years of age** | **Vision test** |
| --- | --- |
| 2-4 | Kay Pictures (crowded Thompson 4m chart, if possible, single if developmental stage / concordance is an obstacle)  If not successful, Cardiff card acuity cards to be used |
| 4+ | Crowded LogMAR (Thompson 4m chart) |

Best achieved uniocular vision with correction (if needed) is recorded, and correction / vision test method used is also recorded
