# Supplementary material for: UVESCREEN1: A randomised feasibility study of imaging-based uveitis screening for children with juvenile idiopathic arthritis- Study Protocol
Source: PLoS One. 2025 Feb 12;20(2):e0316410. doi: 10.1371/journal.pone.0316410 (PMC11819525; doi:10.1371/journal.pone.0316410)
Supplement: Supplemental material 3 — (DOCX) [file pone.0316410.s003.docx]

**Supplemental materials 3- Consent forms**

**CHILD ASSENT FORM**

**Title of Project:  *UVESCREEN1 study***

Child (or if unable, parent on their behalf)/young person to circle all they agree with:

Has somebody explained the project to you Yes / No

Do you understand what this project is about? Yes / No

Have you asked all the questions you want? Yes / No

Have you had your questions answered in a way you understand? Yes / No

Do you understand it’s ok to stop taking part at any time? Yes / No

Are you happy to take part? Yes / No

**If any** **answers are ‘no’ or you don’t want to take part, don’t sign your name!**

**If you do** **want to take part, you can write your name below**

_ Your name Date

The person who explained this project to you needs to sign too:

|  |  |  | ________________ |
| --- | --- | --- | --- |
| Print Name |  | Date | Sign |

**Thank you for your help!**

A randomised feasibility study of imaging-based uveitis screening for children with juvenile idiopathic arthritis

**Parent/Guardian Consent Form (STUDY TEAM COPY*)**

***for storage in locked cabinet within secure clinical research office**

| To be completed by the Parent/Guardian: | |
| --- | --- |
| Once you have read and understood each statement **please enter your initials** in each box. | Initial |
| 1. I have read and understood the information sheet (PIS dated 02/10/2024, V3) for this study. I have had the opportunity to ask questions and have had these answered satisfactorily. |  |
| 1. I understand that participation is voluntary and that I am free to withdraw my child from the study at any time, without giving a reason, and without their care or legal rights being affected. However, I understand that the study team may need to continue to collect some limited information for safety reasons. |  |
| 1. I agree for my child to participate in the above study. |  |
| 1. I understand that relevant sections of my child’s medical notes and any data collected during the study may be looked at by authorised individuals from the central study team and representatives of the Sponsor, regulatory authorities and the local NHS Trust. I give permission for these individuals to have access to my child’s records and data. |  |
| 1. I agree to my child’s GP and local clinician(s) being informed of their participation in the study. |  |
| 1. I understand that a copy of this consent form and my child’s personal data will be held at my hospital |  |
| 1. I understand that my child’s personal data will be archived in a confidential manner for 25 years from the end of the study. |  |
| 1. I agree to allow information and data or results arising from this study to be used in future healthcare and/or medical research in the UK or abroad, providing mine and my child’s confidentiality is maintained. |  |
| 1. I agree to the recording of interviews of me and my child if we are invited to take part in the study interviews |  |
| **The statements below are optional (your child can still take part in the study even if you do not wish to agree to these):** |  |
| 1. I consent for my and my child’s participation in an up to 1 hour interview at the end of the study. |  |
| 1. I agree that I or my child may be contacted in the future in relation to this or other related studies.   (if you agree to this statement provide your details below):   \| Telephone number: \|  \|  \|  \|  \|  \|  \|  \|  \|  \|  \|  \|  \| \| --- \| --- \| --- \| --- \| --- \| --- \| --- \| --- \| --- \| --- \| --- \| --- \| --- \| \| Email address: \|  \| \| \| \| \| \| \| \| \| \| \| \| |  |
