# Supplementary material for: UVESCREEN1: A randomised feasibility study of imaging-based uveitis screening for children with juvenile idiopathic arthritis- Study Protocol
Source: PLoS One. 2025 Feb 12;20(2):e0316410. doi: 10.1371/journal.pone.0316410 (PMC11819525; doi:10.1371/journal.pone.0316410)
Supplement: Attachment — Submitted filename: Reviewer responses.docx [file pone.0316410.s004.docx]

*1) Please upload a copy of the original protocol submitted to the IRB as a Supporting Information file*

This has been submitted

*2) Please provide additional details regarding consent. In the Methods section, you specify that "Informed consent will be sought by a member of the research team within a week of being given the study documentation", but you do not explicitly state who will be asked to provide consent. Also, you specify later in your manuscript that "all participants will provide written or electronic informed consent." As your participants are all aged 2-12 years old, please specify who will be approached to provide consent for the participating children.*

We have clarified that the parent will be approached to provide consent (page 11, lines 225-227 and page 14, line 308).

*3) Please provide more detail regarding the proposed statistical analysis plan.*

On page 13, lines 276-284, we have now reported that: ‘Within this feasibility trial, effect sizes (of ‘discrimination’ of disease) and variability in outcomes will be estimated with 95% confidence intervals to inform the sample size calculations for a full-scale trial. Efficacy and safety will be analysed according to randomisation group set, which will include all patients who underwent randomisation and who had recorded outcomes (positive detection) during the first 12 months or who completed at least 12 months of surveillance examinations. Missing outcomes (due to attrition) will be considered as an adverse outcome. We will compare the two study arms groups using two-by-two chi-square tests for equality of proportions for disease detection / adverse outcomes within the OCT arm as compared with the ‘gold standard’ slit lamp examination group.’
